# mTORC1 Signaling is a Critical Regulator of Postnatal Tendon Development

**DOI:** 10.1038/s41598-017-17384-0

**Published:** 2017-12-07

**Authors:** Joohyun Lim, Elda Munivez, Ming-Ming Jiang, I-Wen Song, Francis Gannon, Douglas R. Keene, Ronen Schweitzer, Brendan H. Lee, Kyu Sang Joeng

**Affiliations:** 1 Department of Molecular and Human Genetics, Baylor College of Medicine, One Baylor Plaza, Houston, TX 77030 USA; 2 Department of Pathology, Baylor College of Medicine, One Baylor Plaza, Houston, TX 77030 USA; 3 Research Center, Shriners Hospital for Children, Portland, OR 97239 USA

## Abstract

Tendons transmit contractile forces between musculoskeletal tissues. Whereas the biomechanical properties of tendons have been studied extensively, the molecular mechanisms regulating postnatal tendon development are not well understood. Here we examine the role of mTORC1 signaling in postnatal tendon development using mouse genetic approaches. Loss of mTORC1 signaling by removal of *Raptor* in tendons caused severe tendon defects postnatally, including decreased tendon thickness, indicating that mTORC1 is necessary for postnatal tendon development. By contrast, activation of mTORC1 signaling in tendons increased tendon cell numbers and proliferation. In addition, *Tsc1* conditional knockout mice presented severely disorganized collagen fibers and neovascularization in the tendon midsubstance. Interestingly, collagen fibril diameter was significantly reduced in both *Raptor* and *Tsc1* conditional knockout mice, albeit with variations in severity. We performed RNA-seq analysis using Achilles tendons to investigate the molecular changes underlying these tendon phenotypes. *Raptor* conditional knockout mice showed decreased extracellular matrix (ECM) structure-related gene expression, whereas *Tsc1* conditional knockout mice exhibited changes in genes regulating TGF-β/BMP/FGF signaling, as well as in genes controlling ECM structure and disassembly. Collectively, our studies suggest that maintaining physiological levels of mTORC1 signaling is essential for postnatal tendon development and maturation.

## Introduction

The tendon is a fibrous connective tissue that is essential for transmitting mechanical forces from muscle to bone. Chronic tendinopathy and tendon rupture are common tendon-related pathological conditions that cause disability and pain, creating a significant healthcare burden^1^. Currently, the development of effective medical interventions for tendinopathy and tendon repair is relatively limited^1,2^. In this regard, understanding the mechanistic basis of tendon development may provide insights to identify novel targets for clinical intervention. However, the cellular and molecular mechanisms regulating tendon development and homeostasis are not fully understood.

Tendon progenitors arise during embryonic development through induction by fibroblast growth factor (FGF) and transforming growth factor β (TGFβ) signaling^3,4^. Tendon progenitors are marked by the expression of *Scleraxis* (*Scx*), a basic helix-loop-helix (bHLH) transcription factor that is critical for tendon differentiation^5,6^. In addition, *Mohawk* (*Mkx*), an atypical homeobox transcription factor, has been shown to be essential for postnatal tendon maturation^7–9^. Early progenitors of the enthesis, the tendon-to-bone insertion site, are marked by both *Scx* and *Sox9* expression^10^. On the other hand, fibrocartilage cells of the enthesis originate from a unique population of *Hedgehog* (*Hh*)-responsive cells, and active Hh signaling may be involved in enthesis development and healing^11–17^. Furthermore, recent studies have identified progenitor populations that contribute to tendon growth and healing at various developmental stages^18–20^.

Tendons are largely composed of type I collagen in addition to various proteoglycans, glycoproteins, and minor collagens^2,21^. In postnatal development, tendons undergo a dramatic transition from predominantly small fibrils (35–40 nm) to a heterogeneous group of fibrils that vary widely in diameter (35–400 nm)^7,8,22^. This transition involves a complex process in which small collagen fibrils undergo lateral fusion to form thicker fibrils^21,22^. Mutations in genes that regulate this process, including small leucine-rich proteoglycans (SLRPs) such as *Decorin* (*Dcn*), *Fibromodulin* (*Fmod*), and *Lumican* (*Lum*), can cause defects in tendon maturation^23–26^.

The mechanistic target of the rapamycin complex 1 (mTORC1) signaling pathway regulates a wide range of biological processes, including cellular metabolism, growth, proliferation, and survival^27^. The mTORC1 protein complex consists of multiple components such as the serine/threonine kinase mTOR and the regulatory-associated protein of mTOR (Raptor). Loss of *Raptor* results in specific inactivation of mTORC1 signaling. This pathway is known to mediate multiple signaling cascades that regulate the tuberous sclerosis complex (TSC) comprising TSC1 and TSC2, which negatively regulates mTORC1 by inhibiting the small GTPase Rheb. Importantly, loss of either *Tsc1* or *Tsc2* leads to activation of mTORC1 signaling^28^, and this approach has been used to show the role of mTORC1 signaling in bone and cartilage development^29,30^. In the context of the tendon, recent studies showed that rapamycin, an inhibitor of mTORC1 activity, can attenuate age-associated changes in tendons^31,32^. However, the specific role of mTORC1 signaling in tendon development and homeostasis is not known.

In this study, we investigated the function of mTORC1 in tendon development by conditionally removing *Raptor* or *Tsc1*, either to inhibit or activate mTORC1 signaling, respectively, in tendon cells. Loss of mTORC1 activity by *Raptor* deletion markedly decreased tendon thickness while maintaining tendon cell number, suggesting defects in tendon maturation. By contrast, activation of mTORC1 signaling in tendon cells by *Tsc1* deletion did not affect tendon thickness but increased tendon cell number and proliferation. In addition, loss of *Tsc1* in tendon cells was associated with disorganized collagen fibers and neovascularization. Both loss- and gain-of-function mouse models exhibited severe defects in collagen fibril maturation, suggesting that precise regulation of mTORC1 signaling is critical for collagen fibrillogenesis. Overall, our studies reveal a critical role for mTORC1 signaling in regulating postnatal tendon development.

## Results

### Loss of mTORC1 signaling impairs postnatal tendon maturation

To understand the function of mTORC1 signaling in tendon development, we conditionally ablated *Raptor* in tendons using the *Scx-Cre* mouse line. *Scx-Cre; Raptor* ^*f/f*^ mice had no obvious phenotype at birth, but began showing mild curls in the tail at 1 week of age. The curly tails were clearly displayed at 1 month of age in *Scx-Cre; Raptor* ^*f/f*^ mice compared to littermate controls (Supplementary Fig. S1A). To characterize the tendon phenotype, we examined tendons from wild-type and *Scx-Cre; Raptor* ^*f/f*^ mice at 1 month of age. Wild-type tendons were white and visibly distinct compared to the adjacent muscle and cartilage, but tendons from *Scx-Cre; Raptor* ^*f/f*^ mice appeared translucent and indistinguishable from the surrounding connective tissues (Supplementary Fig. S1B–D, arrow indicates each tendon), suggesting severe tendon abnormalities.

**Figure 1:**
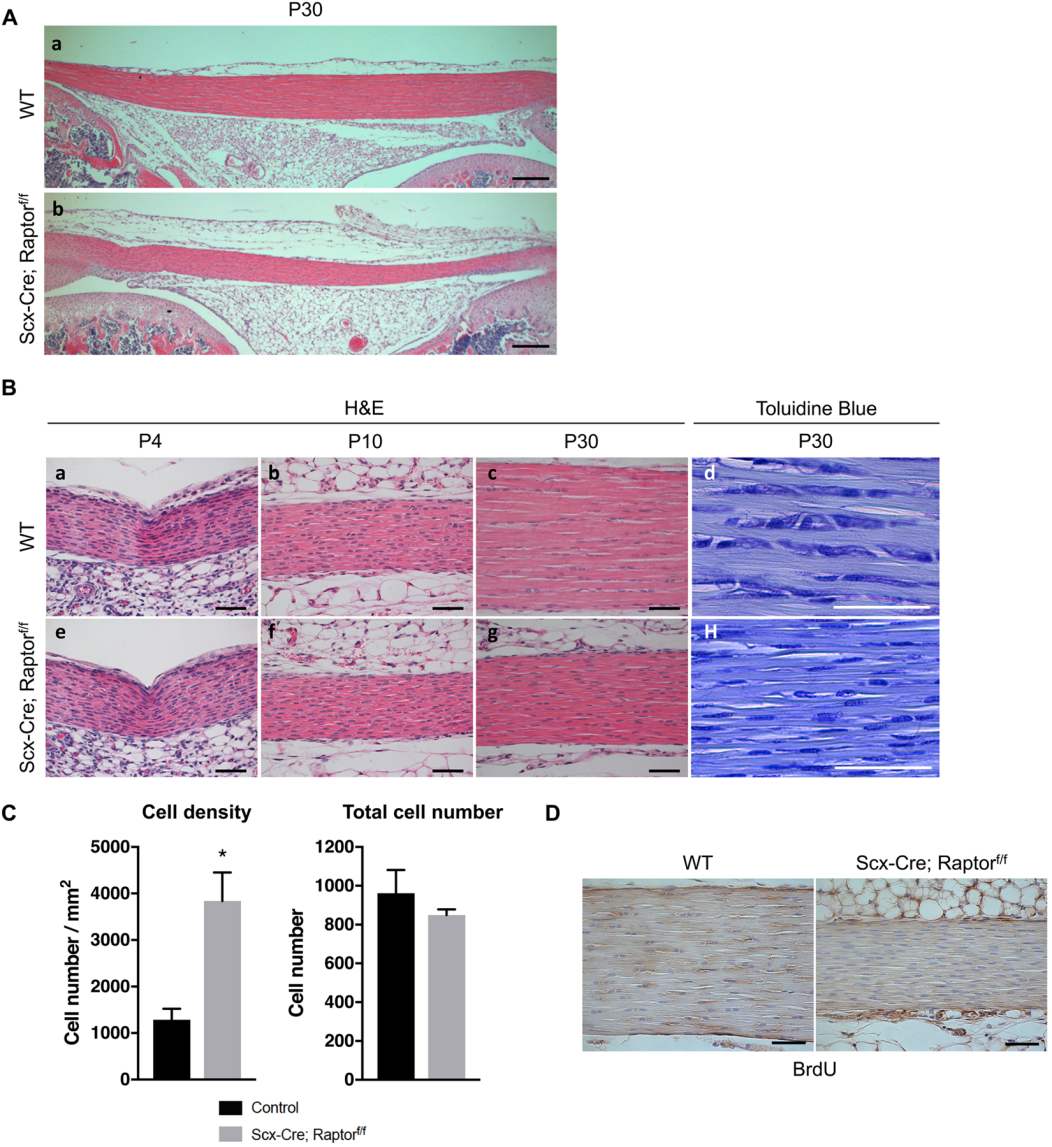
Loss of mTORC1 signaling impairs postnatal maturation of patellar tendons. (**A**) Hematoxylin and Eosin (H&E) staining of patellar tendons from wild-type (WT) (a) and *Scx-Cre; Raptor* ^*f/f*^ littermates (b) at postnatal day 30 (P30). Scale bars indicate 200 μm. (**B**) H&E and toluidine blue-stained sections of patellar tendons from WT (a–d) and *Scx-Cre; Raptor* ^*f/f*^ littermates (e–h) at postnatal day 4 (P4), day 10 (P10), and day 30 (P30). Scale bars indicate 50 μm. (**C**) Quantification of cell density and total cell number using H&E and toluidine blue-stained sections of patellar tendons (n = 3, *p < *0*.*05*). (**D**) BrdU staining of patellar tendons from WT and *Scx-Cre; Raptor* ^*f/f*^ littermates at P30. Scale bars indicate 50 μm.

To further assess the tendon defects in *Scx-Cre; Raptor* ^*f/f*^ mice, we performed histological analysis of patellar and Achilles tendons. Between birth and 1 month of age, wild-type patellar tendons showed increased thickness and reduced cell density (Fig. 1A (a) and Fig. 1B (a–c)), which are consistent with previous reports^22^. In stark contrast, *Scx-Cre; Raptor* ^*f/f*^ mice exhibited a dramatic decrease in tendon thickness at 1 month of age when compared with wild-type mice (Fig. 1A (a,b) and Fig. 1B (c,g)), concomitant with a significant increase in tendon cell density (Fig. 1B (c,d,g,h) and Fig. 1C). Interestingly, tendon thickness and histology appeared normal at earlier stages (Fig. 1B (a,b,e,f)), indicating impaired postnatal tendon maturation in *Scx-Cre; Raptor* ^*f/f*^ mice. Quantification results showed that patellar tendons of *Scx-Cre; Raptor* ^*f/f*^ mice did not exhibit changes in total cell number (Fig. 1C). No BrdU-positive cells were found in either wild-type or *Scx-Cre; Raptor* ^*f/f*^ mice (Fig. 1D), and proliferating chondrocytes were BrdU-positive as expected, serving as internal controls (Supplementary Fig. S2A). Taken together, these data suggest that the increased tendon cell density in *Scx-Cre; Raptor* ^*f/f*^ mice is likely due to decreased tendon thickness (i.e. matrix content).

**Figure 2:**
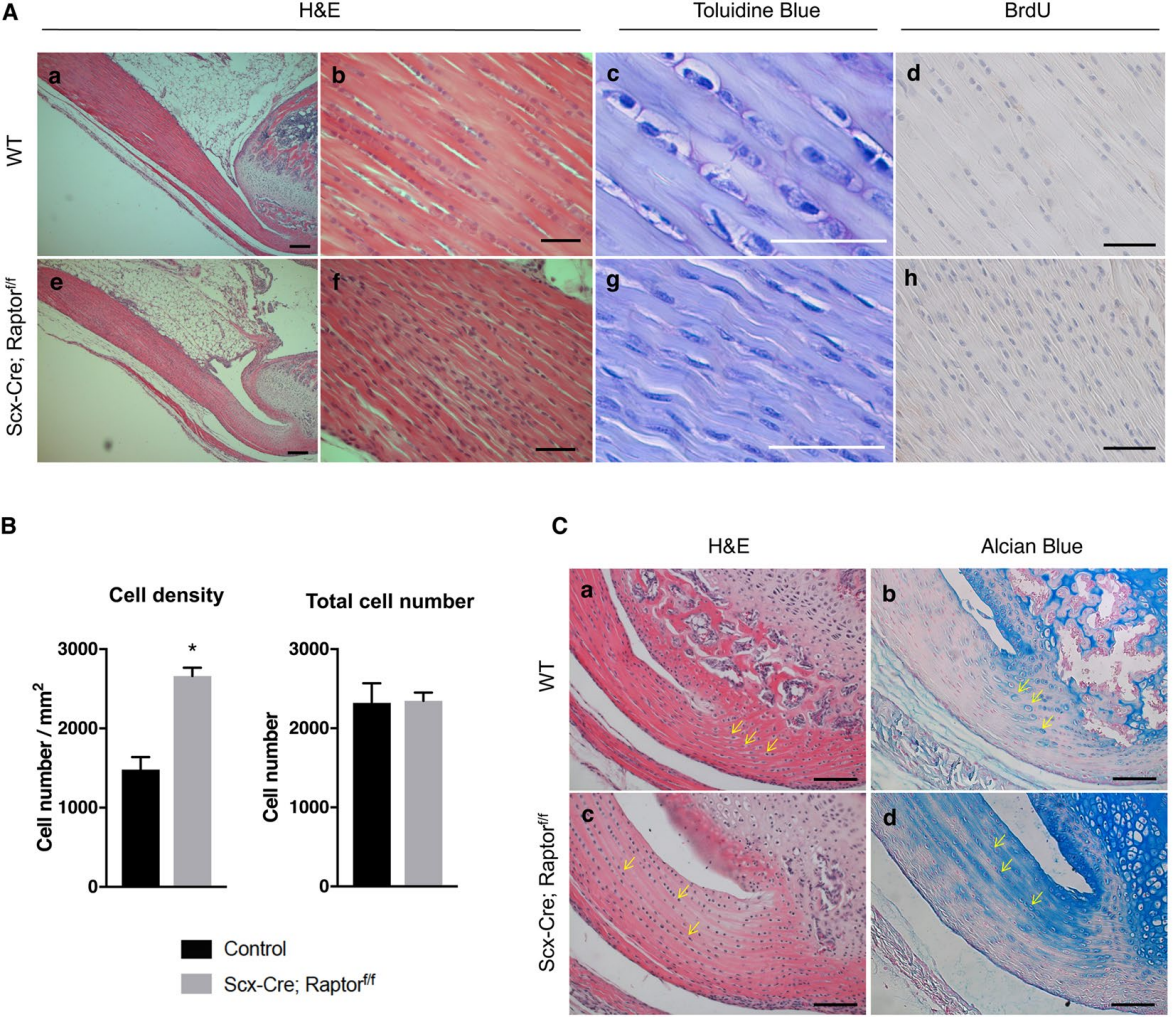
Loss of mTORC1 signaling causes abnormalities in Achilles tendons. (**A**) Histology of Achilles tendons from wild-type (WT) (a-d) and *Scx-Cre; Raptor* ^*f/f*^ littermates (e–h) at postnatal day 30 (P30). Low magnification (5X) image of Hematoxylin and Eosin (H&E) staining of Achilles tendon (a and e). High magnification image (40X) of H&E (b and f), toluidine blue (c and g), and BrdU (d and h) staining of the mid-substance of Achilles tendons. Scale bars in a and e indicate 200 μm, and all other scale bars indicate 50 μm. (**B**) Quantification of cell density and total cell number using H&E-stained sections of Achilles tendons (n = 3, *p < *0*.*05*). (**C**) H&E staining of the enthesis of the Achilles tendons from WT (a) and *Scx-Cre; Raptor* ^*f/f*^ littermates (c) at P30. Alcian blue staining of the enthesis of Achilles tendons from WT (b) and *Scx-Cre; Raptor* ^*f/f*^ littermates (d) at P30. Yellow arrows indicate representative fibrocartilage cells. Scale bars indicate 100 μm.

Consistent with the histological features observed in the patellar tendon, the mid-substance of the Achilles tendon also showed decreased tendon thickness (Fig. 2A (a and e)) and increased cell density (Fig. 2A (b,c,f,g)) in 1 month old *Scx-Cre; Raptor* ^*f/f*^ mice. Quantification results confirmed that *Scx-Cre; Raptor* ^*f/f*^ mice have increased cell density and no changes in total cell number (Fig. 2B). In addition, no BrdU-positive cells were found in either wild-type or *Scx-Cre; Raptor* ^*f/f*^ mice (Fig. 2A (d and h)).

Fibrocartilage cells reside in the enthesis of wild-type mice (Fig. 2C (a), yellow arrows) and can be easily distinguished from tenocytes based on cell morphology. Interestingly, *Scx-Cre; Raptor* ^*f/f*^ mice developed ectopic fibrocartilage-like cells in regions distal to the enthesis (Fig. 2C (c), yellow arrows). The location of the abnormal fibrocartilage-like cells in the enthesis was associated with ectopic Alcian blue staining in the Achilles tendon of *Scx-Cre; Raptor* ^*f/f*^ mice (Fig. 2C (d)), while Alcian blue staining was restricted to the enthesis in control mice (Fig. 2C (b)). The ectopic fibrocartilage-like cells in *Scx-Cre; Raptor* ^*f/f*^ mice were also observed in patellar tendons, albeit to a lesser extent (Supplementary Fig. S2B). Interestingly, type II collagen expression remained restricted to the enthesis in both wild-type and *Scx-Cre; Raptor* ^*f/f*^ mice (Supplementary Fig. S2C). Collectively, these results suggest that mTORC1 signaling is essential for postnatal maturation of both patellar and Achilles tendons.

### Gain of mTORC1 signaling induces tendon abnormalities

To further understand the function of mTORC1 in tendon development, we generated *Scx-Cre; Tsc1* ^*f/f*^ mice to activate mTORC1 signaling in tendon cells. *Scx-Cre; Tsc1* ^*f/f*^ mice had no obvious phenotype at birth, but showed reduced body size around 3 weeks of age. *Scx-Cre; Tsc1* ^*f/f*^ mice displayed increased mortality starting at 1 month, and few animals lived until 2 months of age. A thorough examination revealed that *Scx-Cre; Tsc1* ^*f/f*^ mice had polycystic kidney disease (PKD) (data not shown). The development of PKD could be directly due to activation of mTORC1 in the kidney, as *ScxGFP* reporter activity was previously detected in mouse embryonic kidney^33^ and mTORC1 activation in the kidney has been shown to cause PKD^34^. Therefore, the postnatal lethality in *Scx-Cre; Tsc1* ^*f/f*^ mice is likely caused by renal failure.

**Figure 3:**
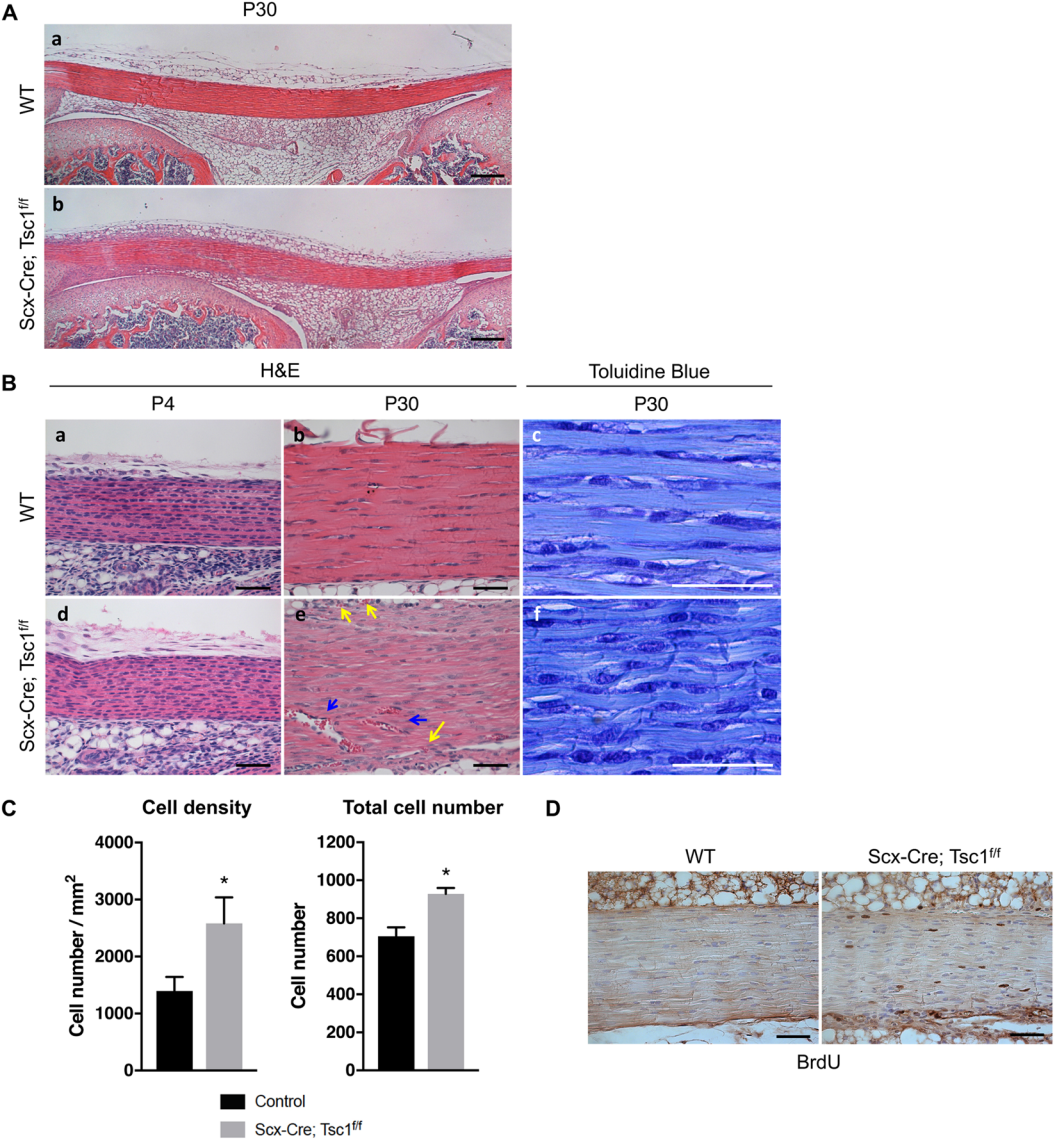
Gain of mTORC1 signaling induces tendon abnormalities. (**A**) Hematoxylin and Eosin (H&E) staining of patellar tendons from wild-type (WT) (a) and *Scx-Cre; Tsc1* ^*f/f*^ littermates (b) at P30. Scale bars indicate 200 μm. (**B**) H&E and toluidine blue-stained sections of patellar tendons from WT (a–c) and *Scx-Cre; Tsc1* ^*f/f*^ littermates (d–f) at P4 and P30. Scale bars indicate 50 μm. Arrows indicate vascularization in the midsubstance (blue arrows), as well as in regions between the patellar tendon and surrounding fat pads (yellow arrows) (**C**) Quantification of cell density and total cell number using toluidine blue-stained sections of patellar tendons (n = 3, *p < *0*.*05*). (**D**) BrdU staining of patellar tendons from WT and *Scx-Cre; Tsc1* ^*f/f*^ littermates at P30. Scale bars indicate 50 μm.

At 1 month of age, *Scx-Cre; Tsc1* ^*f/f*^ mice consistently developed kinks at the distal end of the tail (Supplementary Fig. S3A). The majority of tendons in *Scx-Cre; Tsc1* ^*f/f*^ mice were pale-white in color compared with those of wild-type littermates, but still distinct relative to the adjacent connective tissue (Supplementary Fig. S3B–D, arrow indicates each tendon). Histological analysis showed no obvious changes in tendon thickness at 1 month of age (Fig. 3A (a,b)). Whereas tendon histology was relatively normal in neonates (Fig. 3B (a,d)), the patellar tendons of *Scx-Cre; Tsc1* ^*f/f*^ mice at 1 month of age exhibited disorganized tenocytes and collagen fibers relative to littermate controls (Fig. 3B (b,c,e,f)). *Scx-Cre; Tsc1* ^*f/f*^ mice also showed increased number of red blood cells in the midsubstance (Fig. 3B (e), blue arrows) as well as in regions between the patellar tendon and surrounding fat pads (Fig. 3B (e), yellow arrows), indicating neovascularization. Furthermore, both the total tendon cell number and density were significantly increased in the patellar tendons of *Scx-Cre; Tsc1* ^*f/f*^ mice, which coincided with an increase in tendon cell proliferation (Fig. 3C and D).

**Figure 4:**
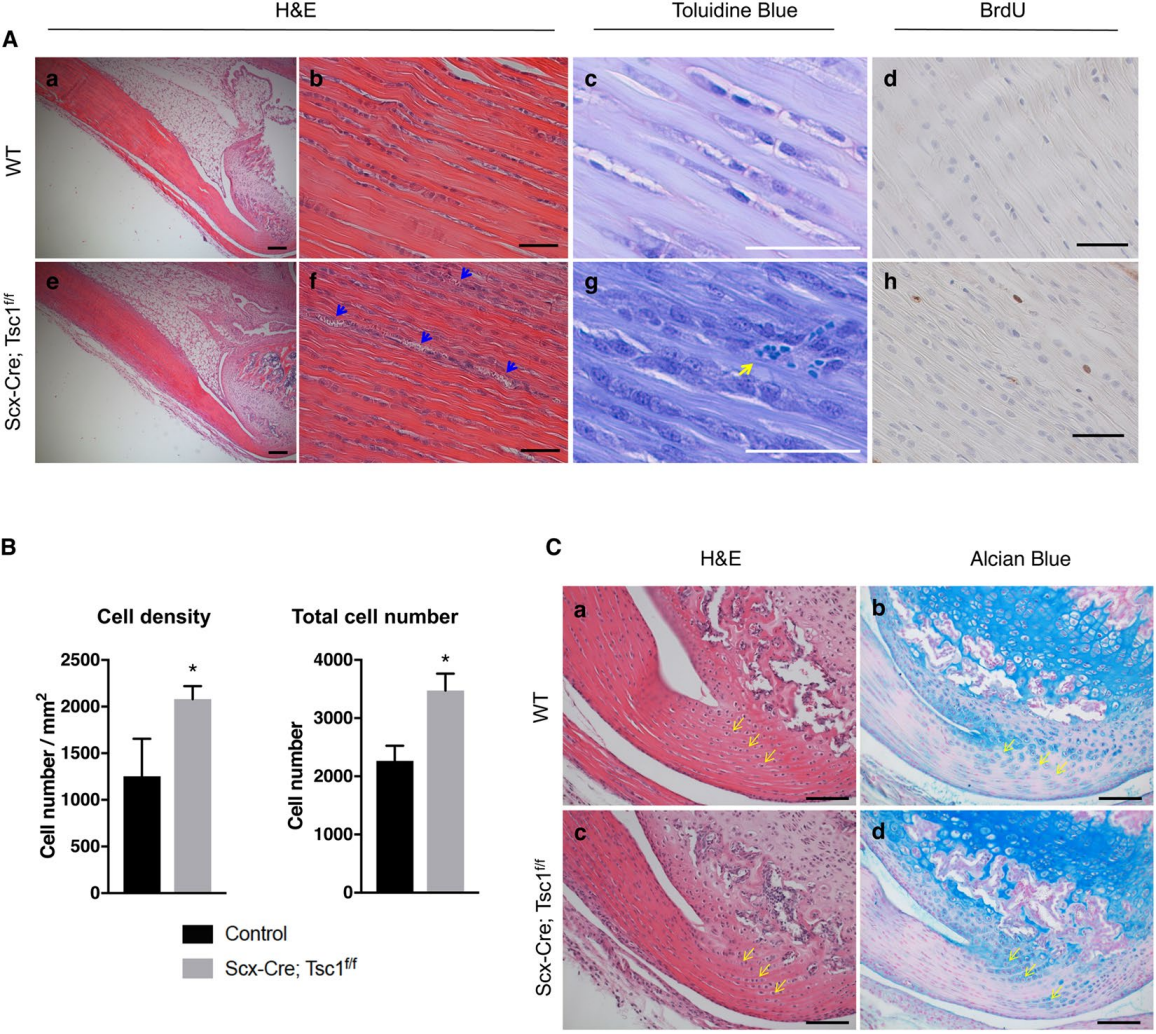
Gain of mTORC1 signaling induces abnormalities in Achilles tendons. (**A**) Histology of Achilles tendons from wild-type (WT) (a-d) and *Scx-Cre; Tsc1* ^*f/f*^ littermates (e–h) at postnatal day 30 (P30). Low magnification (5X) image of Hematoxylin and Eosin (H&E) staining of Achilles tendons (a and e). High magnification image (40X) of H&E (b and f), toluidine blue (c and g), and BrdU (d and h) staining of the midsubstance of Achilles tendons. Blue arrows and yellow arrow indicate vascularization. Scale bars in a and e indicate 200 μm, and all other scale bars indicate 50 μm. (**B**) Quantification of cell density and total cell number using H&E-stained sections of Achilles tendons (n = 3, *p < *0*.*05*). (**C**) H&E staining of the enthesis of the Achilles tendon from WT (a) and *Scx-Cre; Tsc1* ^*f/f*^ littermates (c) at P30. Alcian blue staining of the enthesis of Achilles tendons from WT (b) and *Scx-Cre; Tsc1* ^*f/f*^ littermates (d) at P30. Yellow arrows indicate representative fibrocartilage cells. Scale bars indicate 100 μm.

The Achilles tendon exhibited histological phenotypes similar to those observed in the patellar tendon of *Scx-Cre; Tsc1* ^*f/f*^ mice. First, no obvious changes were observed in tendon thickness at 1 month of age (Fig. 4A (a,e)). Second, the Achilles tendons of *Scx-Cre; Tsc1* ^*f/f*^ mice at 1 month of age exhibited disorganized tenocytes and collagen fibers relative to littermate controls (Fig. 4A (b,c,f,g)). Third, *Scx-Cre; Tsc1* ^*f/f*^ mice also showed increased number of red blood cells in the midsubstance (Fig. 4A (f,g), blue and yellow arrows), indicating neovascularization. Finally, both total tendon cell number and cell density were significantly increased in the Achilles tendons of *Scx-Cre; Tsc1* ^*f/f*^ mice, which coincided with an increase in tendon cell proliferation (Fig. 4A and B (d,h)). Unlike the loss-of-function mouse model, we did not observe an increase in fibrocartilage-like cells or ectopic Alcian blue staining near the enthesis in either patellar or Achilles tendons of *Scx-Cre; Tsc1* ^*f/f*^ mice (Fig. 4C (a–d), and Supplementary Fig. S4B). Overall, these data suggest that maintaining physiological levels of mTORC1 signaling is required for proper postnatal tendon development and maturation.

### Altered mTORC1 signaling causes impaired collagen fibrillogenesis during postnatal tendon development

**Figure 5:**
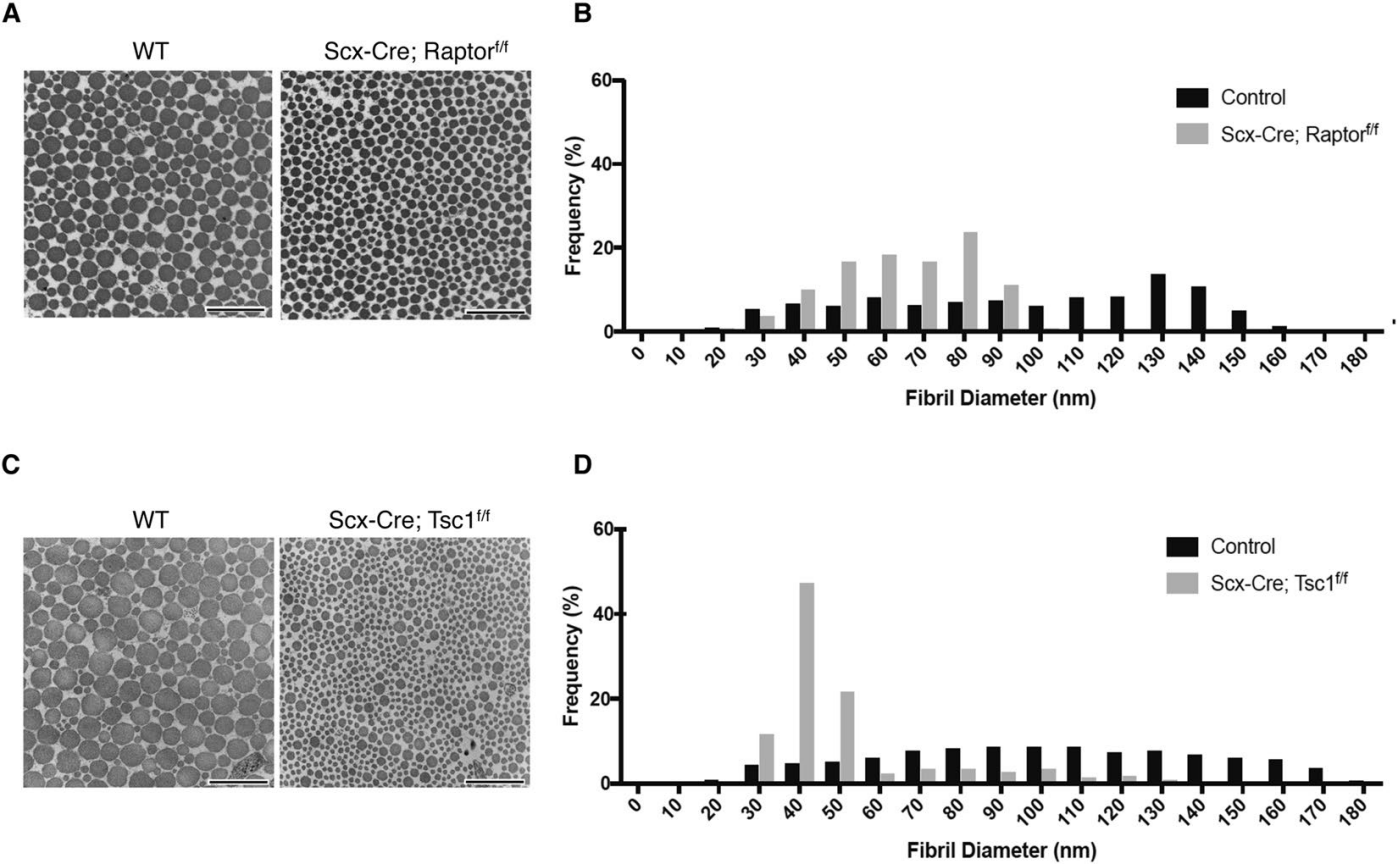
Altered mTORC1 signaling significantly affects collagen fibrillogenesis. (**A**) Transverse section transmission electron microscopy (TEM) image of Achilles tendons of wild-type (WT) and *Scx-Cre; Raptor* ^*f/f*^ littermates at P30. (**B**) Quantification of collagen fibril diameter using TEM images from Achilles tendons of wild-type and *Scx-Cre; Raptor* ^*f/f*^ littermates at P30 (n = 3). (**C**) Transverse section TEM image of Achilles tendons of WT and *Scx-Cre; Tsc1* ^*f/f*^ littermates at P30. (**D**) Quantification of collagen fibril diameter using TEM images from Achilles tendons of wild-type and *Scx-Cre; Tsc1* ^*f/f*^ littermates at P30 (n = 3).

To investigate the function of mTORC1 in collagen fibrillogenesis, we performed transmission electron microscopy (TEM) using Achilles tendons of both loss- and gain-of-function mouse models. TEM imaging experiments showed that *Scx-Cre; Raptor* ^*f/f*^ (loss-of-function) mice had a significantly smaller collagen fibril diameter compared with wild-type littermates (Fig. 5A). Specifically, the distribution of collagen fibril diameter ranged from 20 to 90 nm in *Scx-Cre; Raptor* ^*f/f*^ mice, whereas wild-type mice exhibited a wider distribution of fibril diameter ranging from 20 to 180 nm (Fig. 5B). *Scx-Cre; Tsc1* ^*f/f*^ (gain-of-function) mice also had significantly smaller collagen fibril diameter compared with wild-type littermates (Fig. 5C). However, unlike *Scx-Cre; Raptor* ^*f/f*^ mice, the distribution of fibril diameters in *Scx-Cre; Tsc1* ^*f/f*^ mice was predominantly enriched between 30 and 50 nm (80% of fibrils) but showed greater heterogeneity with larger fibrils of up to 130 nm (Fig. 5D). Overall, the data from both gain- and loss-of-function studies suggest that mTORC1 signaling is a critical regulator of postnatal collagen fibril maturation.

### Transcriptome analysis of tendons from loss- and gain-of-function mouse models of mTORC1

To investigate the molecular changes underlying tendon phenotypes, we performed RNA-seq with RNA isolated from Achilles tendons of both loss- and gain-of-function mouse models at 1 month of age. To determine global changes in differentially expressed genes, we performed Ingenuity Pathway Analysis (IPA, Qiagen, Germany). As expected, IPA results showed that differentially expressed genes were highly enriched in mTORC1-related pathways in loss- and gain-of-function mouse models, including “Regulation of eIF4 and p70S6K Signaling”, “mTOR Signaling”, “Oxidative Phosphorylation”, and “Mitochondrial Dysfunction” (Supplementary Fig. S5, Supplementary Table 1, and Supplementary Table 2).

**Figure 6:**
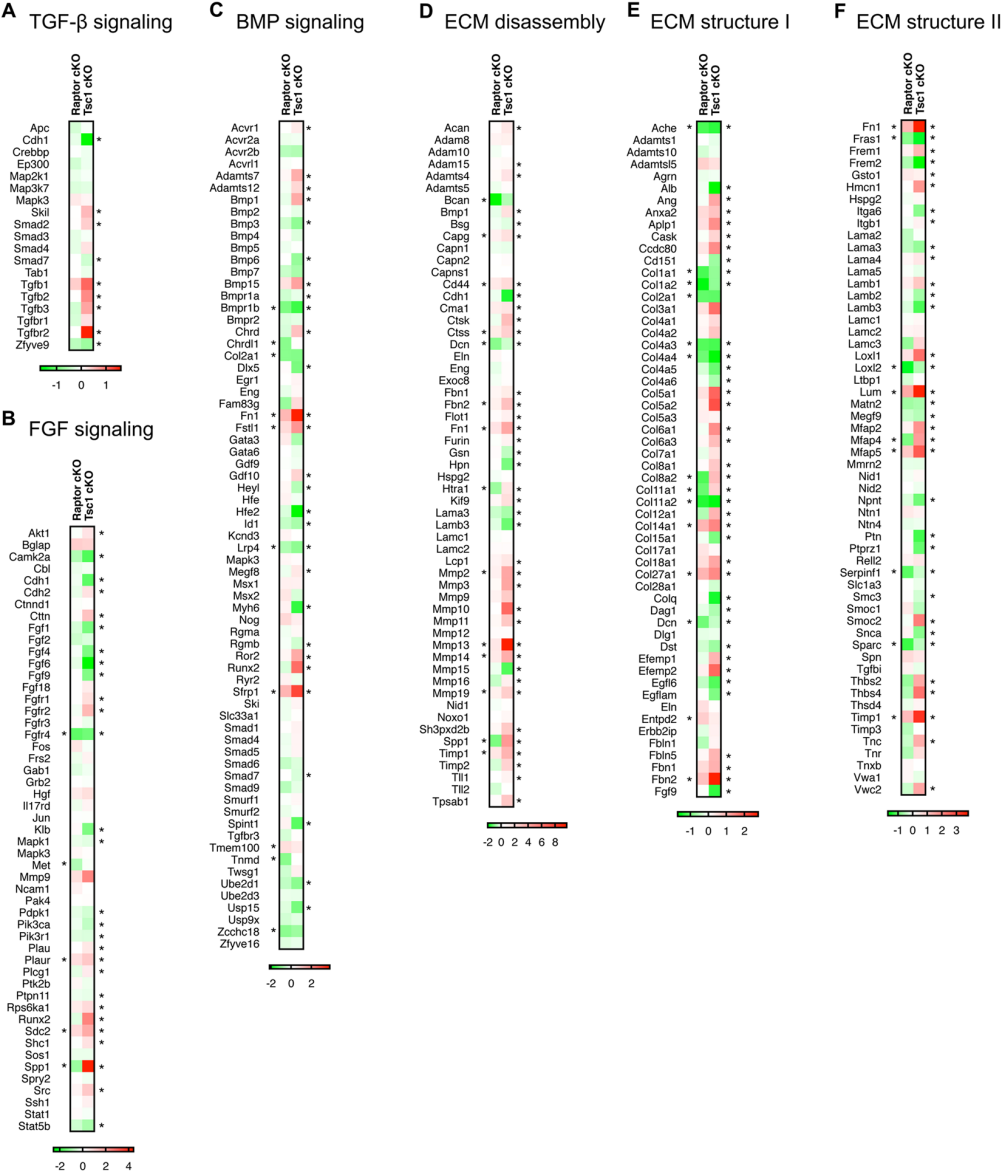
Transcriptome analysis of tendons from loss- and gain-of-function mouse models of mTORC1. The Gene Set Enrichment Analysis (GSEA) database was used to identify and plot Log2 fold change in RNA-seq data from Achilles tendons of Raptor conditional knockout (cKO) mice (control vs. *Scx-Cre; Raptor* ^*f/f*^) or Tsc1 cKO (control vs. *Scx-Cre; Tsc1* ^*f/f*^) mice in (**A**) TGF-β signaling, (**B**) FGF signaling, (**C**) BMP signaling, (**D**) extracellular matrix disassembly, and (**E**,**F**) extracellular matrix structure (n = 3, *p < 0.05).

To further understand the molecular changes in both mouse models, we first examined whether major signaling pathways involved in tendon development are affected in *Raptor* or *Tsc1* conditional knockout mice. Despite significant changes in tendon morphology, *Scx-Cre; Raptor* ^*f/f*^ mice only showed subtle changes in genes involved in TGF-β/BMP and FGF signaling pathways (Fig. 6A–C, left panels, Supplementary Table 3). By contrast, expression of TGF-β1-3, Tgfbr2, and Smad2 was markedly increased in *Scx-Cre; Tsc1* ^*f/f*^ mice (Fig. 6A, right panel), and genes involved in BMP and FGF signaling were also broadly affected in *Scx-Cre; Tsc1* ^*f/f*^ mice (Fig. 6B and C, right panels). Furthermore, *Tsc1* deletion significantly affected the expression of genes that regulate ECM structure and disassembly, including collagens and Mmp genes, respectively (Fig. 6D–F, right panel, Supplementary Table 3). On the other hand, several collagen genes were predominantly downregulated in *Scx-Cre; Raptor* ^*f/f*^ mice (Fig. 6E, left panel), while only mild changes were observed in genes involved in ECM disassembly (Fig. 6D, left panel). Taken together, these results suggest that the tendon phenotypes of *Raptor* conditional knockout mice are associated with decreases in ECM structure-related gene expression, whereas the tendon phenotypes of *Tsc1* conditional knockout mice are associated with changes in signaling-related gene expression, as well as with those controlling ECM structure and disassembly.

**Figure 7:**
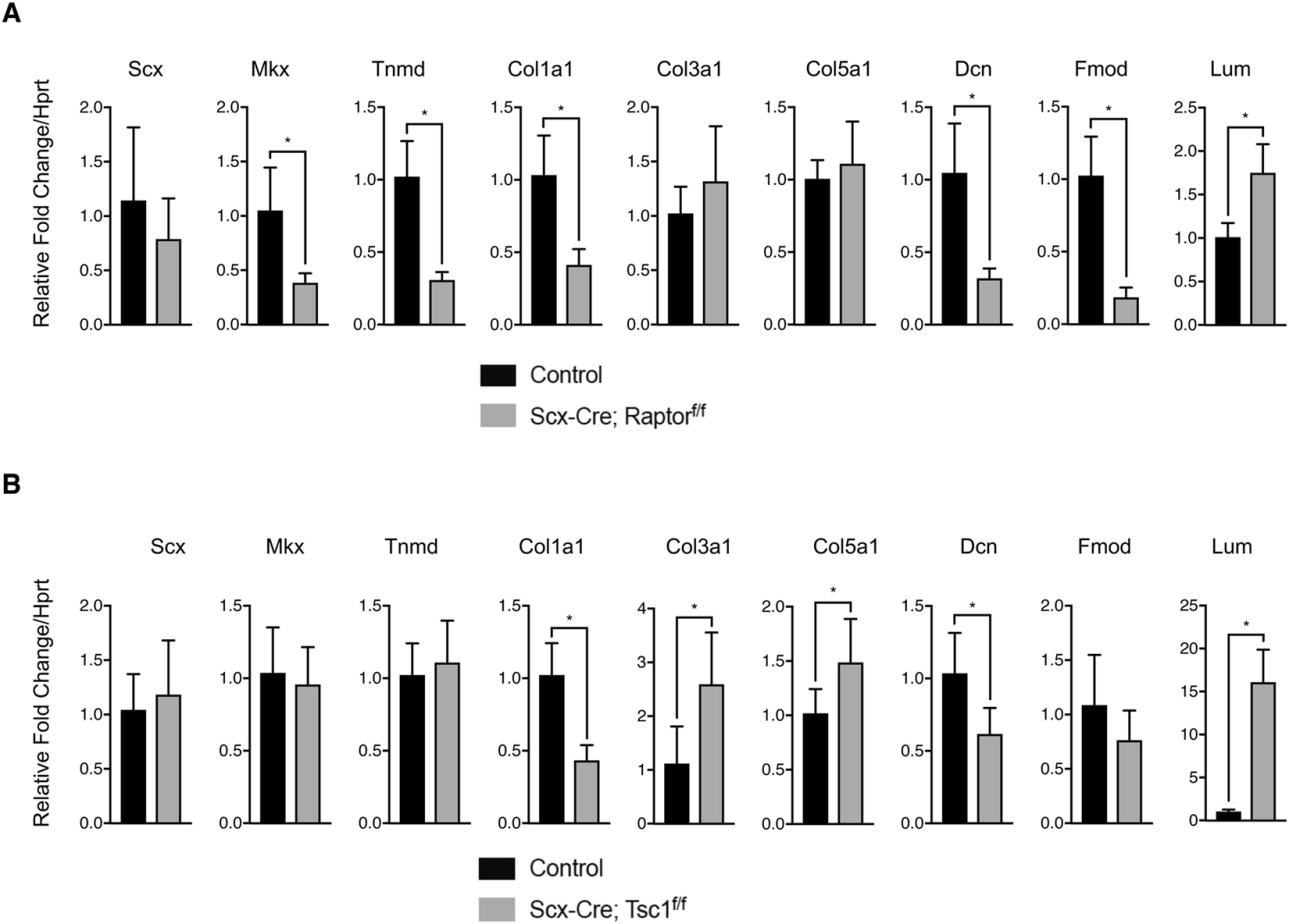
qRT-PCR validation of tendon-related genes from loss- and gain-of-function mouse models of mTORC1. Gene expression of *Scx*, *Mkx*, *Tnmd*, *Col1a1*, *Col3a1*, *Col5a1*, *Dcn*, *Fmod*, and *Lum* shown as relative fold change to *Hprt* in (**A**) loss-of-function (n = 6, *p < 0.05) and (**B**) gain-of-function mouse models of mTORC1 (n = 8, *p < 0.05).

We next examined the expression patterns of genes that are implicated in embryonic and postnatal tendon development. Conditional deletion of *Raptor* did not affect *Scx* expression but significantly reduced *Mkx* and *Tnmd* levels (Fig. 7A). However, activating mTORC1 signaling by *Tsc1* removal did not alter *Scx*, *Mkx* or *Tnmd* levels (Fig. 7B). Mature tendons predominantly consist of type I collagen but also comprise minor quantities of type III and V collagen. Loss of *Raptor* significantly reduced type I collagen expression without affecting type III or V levels (Fig. 7A). Interestingly, *Tsc1* deletion also decreased type I collagen expression but greatly increased both type III and V levels (Fig. 7B). In addition to fibrillar collagens, several SLRPs have been shown to be important for normal collagen fibrillogenesis. Therefore, we next examined the expression of *Dcn*, *Fmod*, and *Lum*. Interestingly, *Dcn* and *Fmod* expression was reduced while *Lum* levels were upregulated in both mTORC1 loss- and gain-of-function mice, although there were noticeable differences in the magnitude of change (Fig. 7A and B). Overall, we speculate that reduced expression of type I collagen and altered expression of SLRPs including *Dcn*, *Fmod* and *Lum* could be associated with aberrations in collagen fibrillogenesis in both mTORC1 loss- and gain-of-function mice.

## Discussion

We have investigated the function of mTORC1 signaling in tendons using mouse genetic approaches. Whereas the loss of mTORC1 signaling decreases tendon thickness in postnatal mice, activation of the pathway causes disorganized fibers, hypercellularity, and neovascularization. Both inhibition and activation of mTORC1 signaling impaired collagen fibrillogenesis in tendons postnatally. Taken together, these data suggest that mTORC1 signaling is necessary for postnatal tendon development while aberrant activation of the pathway may induce pathological conditions in tendon. Furthermore, precise regulation of mTORC1 signaling is required for the proper maturation of collagen fibrils.

Loss of *Raptor* in tendon cells reduced tendon thickness but did not alter tendon cell number or proliferation. Thus, increased cell density in *Raptor* conditional knockout mice is likely secondary to decreased tendon thickness. Because mTORC1 signaling is critical for protein synthesis^28,30^, we speculate that loss of mTORC1 signaling may impair matrix production in tenocytes. Indeed, *Raptor* deletion decreases intercellular distance in tendons, which may reflect a reduction in matrix production. In addition, recent studies have shown that conditional deletion of *Raptor* in mesenchymal progenitors leads to reduced matrix production in chondrocytes owing to lower levels of protein synthesis^30^. Still, further investigation is needed to determine whether mTORC1 signaling is important for protein synthesis in tendon cells.

In contrast to *Raptor* removal, loss of *Tsc1* in tendon cells did not affect tendon thickness but significantly increased tendon cell number and proliferation, which accounts for the increase in cell density. Given that inactivation of the TSC1/2 complex has been shown to cause benign tumors in several tissues^28^, abnormal increases in cell proliferation may partly contribute to tissue pathogenesis in *Tsc1* conditional knockout mice. Consistent with this notion, *Tsc1* conditional knockout mice showed severely disorganized collagen fibers and neovascularization in the tendon midsubstance. Interestingly, these tendon phenotypes shared several common histological features with human tendinopathy^35^. Previous studies have shown that mTORC1 signaling is involved in muscle atrophy and fatty acid infiltration after rotator cuff tears, but its role in tendinopathy has not been investigated^36–38^. RNA-seq results for *Scx-Cre; Tsc1* ^*f/f*^ mice showed overlapping changes in gene expression associated with human tendinopathy including *Mmps*, collagens, and kinases^39^. Further studies will be required to establish the potential involvement of mTORC1 signaling in tendinopathy by using inducible mouse genetic models, and also by evaluating mTORC1 signaling in diseased human tendons.

Although inhibition or activation of mTORC1 signaling in tendons leads to distinct histological phenotypes, collagen fibrillogenesis was significantly impaired in both loss- and gain-of-function mouse models. Despite this similarity, *Scx-Cre; Tsc1* ^*f/f*^ mice displayed a relatively heterogeneous distribution of fibril diameter compared with those of *Scx-Cre; Raptor* ^*f/f*^ mice. *Scx-Cre; Tsc1* ^*f/f*^ mice had more than 80% of collagen fibrils ranging between 30 and 50 nm in diameter, but they also had larger fibrils, up to 130 nm in diameter. On the other hand, *Scx-Cre; Raptor* ^*f/f*^ mice had more than 90% of collagen fibrils between 20 and 90 nm, and no collagen fibrils were bigger than 100 nm. It is worth noting that the distribution patterns of collagen fibril diameter in *Scx-Cre; Raptor* ^*f/f*^ mice resemble those of neonates at P9, while collagen fibril diameter profiles of *Scx-Cre; Tsc1* ^*f/f*^ mice appear similar to those observed in tendons of embryonic stage E18.5 mice^8,22^. Further studies are required to determine whether changes in mTORC1 signaling affect collagen fibrillogenesis at distinct stages of development. Nevertheless, these results suggest that changes in mTORC1 signaling can have significant consequences on collagen fibrillogenesis.

Our RNA-seq experiments revealed unique transcriptional changes in the loss- and gain-of-function mTORC1 mouse models. Interestingly, *Raptor* deletion only had mild effects on cell signaling pathways that regulate tenocyte development, but significantly reduced genes important for ECM structure, including several collagens. Thus, we speculate that mTORC1 primarily regulates tendon cell function through regulation of ECM components. On the other hand, *Tsc1* deletion broadly affected the TGF-β, BMP, and FGF signaling pathways. Notably, TGF-β1-3, Tgfbr2, and Smad2 were significantly upregulated, indicating that TGF-β signaling may be increased in *Tsc1* conditional knockout mice. *Tsc1* removal also significantly induced the expression of several collagens and Mmps, suggesting altered ECM remodeling. Both *Raptor* and *Tsc1* knockout mice showed similar changes in the expression of SLRPs such as *Dcn*, *Fmod*, and *Lum*, which regulate collagen fibril growth through direct interaction with type I collagen^21,23,26^. Specifically, both mouse models showed decreased expression of *Dcn* and *Fmod*, but increased expression of *Lum*. Previous studies have shown that *Lum* expression is increased in tendons of *Fmod*-null mice^25^; thus, it is possible that the altered expression of *Dcn*, *Fmod*, and *Lum* is associated with impaired fibrillogenesis in loss- and gain-of-function mouse models of mTORC1 signaling. qRT-PCR validation of tendon-related genes showed the results largely consistent with the RNA-seq analysis in all genes examined. However, it is worth noting that qRT-PCR experiments did not recapitulate the significant increase in *Scx* expression seen in the RNA-seq of *Tsc1* conditional knockout mice. The RNA-seq of *Scx* showed unusually high variability between samples and aberrant coverage in the intron region, which was not observed for other genes examined. Due to the large sample size and vigorous testing for the qRT-PCR experiments, we conclude that *Scx* expression is unaltered in *Tsc1* conditional knockout mice.

Enthesis rupture and failure of surgical intervention is a common, and yet an unresolved, issue in the field of orthopedic medicine. Interestingly, recent studies have shed light on the presence of Hh-responsive Gli1-positive progenitor cells that are required for postnatal enthesis development and injury repair^11–17^. Still, the molecular mechanisms known to be involved in enthesis repair and regeneration are limited. In our current study, we observed ectopic Alcian blue staining near the enthesis in both patellar and Achilles tendons of *Raptor* conditional knockout mice, which coincided with the appearance of fibrocartilage-like cells. Interestingly, these ectopic fibrocartilage-like cells in *Raptor* conditional knockout mice did not express type II collagen, which suggests that ectopic fibrocartilage-like cells are distinct from the endogenous entheseal fibrocartilage cells. One possible explanation could be that the ectopic induction of fibrocartilage-like cells in *Raptor* conditional knockout mice may be due to an injury response in the tissue. If so, the severity of the injury response may vary depending on the anatomic locations, which likely have distinct mechanical loading properties. Indeed, ectopic Alcian blue-positive staining was stronger in the Achilles relative to patellar tendon, which are thought to experience different mechanical loads. Future studies are warranted to examine changes in tendon function from *Raptor* and *Tsc1* conditional knockout mice and decipher differences in biomechanical properties between anatomically unique regions. Furthermore, lineage-tracing studies will be required to characterize the cellular origin and identity the fibrocartilage-like cells in *Raptor* conditional knockout mice. Determining the mechanism by which these fibrocartilage-like cells are induced will be an interesting future direction of the current study.

## Methods

### Animals

The *Scx-Cre* mouse line was previously described^40^. *Raptor* ^*f/f*^ and *Tsc1* ^*f/f*^ mice were purchased from the Jackson Laboratory (Bar Harbor, ME, USA)^41,42^. All studies were approved by the Institutional Animal Care and Use Committee (IACUC) and Center for Comparative Medicine (CCM) at Baylor College of Medicine (Houston, Texas, USA), and we confirm that all the methods in current studies were performed in accordance with the relevant guidelines and regulations following the IACUC approved protocol.

### Histological analysis

Mouse hindlimbs were collected from 1-month old animals and fixed in 10% neutral buffered formalin overnight at room temperature. Samples were paraffin-embedded and sectioned at 6 µm, following decalcification in 14% (w/v) EDTA (pH 7.4) for 2 weeks with daily solution changes. The paraffin sections of patellar and Achilles tendons were used for Hematoxylin and Eosin (H&E), Picrosirius Red/Alcian Blue, and Toluidine Blue staining. Histological evaluation was blindly performed by 3 independent individuals, including one pathologist.

### RNA-seq

Achilles tendons were excised from the calcaneus, immediately distal to the tendon-muscular-junction. RNA extraction was performed with the Fibrous Connective Tissue kit (Qiagen). Primary mRNA was isolated using Dynabeads Oligo (dT)_25_ magnetic beads (Invitrogen) and fragmented using the NEBNext Magnesium RNA Fragmentation Module (New England Biolabs). Double-stranded cDNA was synthesized with SuperScript Double-Stranded cDNA Synthesis Kit (Invitrogen) to prepare the library templates. The TruSeq RNA Library Preparation Kit (Illumina) was used to generate the RNA-seq library. Illumina NextSeq 500 (loss-of-function study) and Illumina HiSeq 2000 (gain-of-function study) instruments were used to perform sequencing experiments at DNA Link Inc. (CA, San Diego) and at the Human Genome Sequencing Center at Baylor College of Medicine. Raw reads were aligned to the mouse genome NCBI37/mm10 using TopHat. The Mouse genome annotation file was downloaded from the UCSC site (http://genome.ucsc.edu/). The aligned transcripts were then assembled by Cufflinks. After acquiring the final transcriptome through Cuffmerge, the differentially expressed genes were derived using DESeq2. The expression was shown as Base Means, and read counts of genes were normalized with total reads.

### Pathway analysis and gene set plot analysis

Differentially expressed genes deriving through DESeq2 results were used for ingenuity pathway analysis (IPA) analysis (Ingenuity Systems; www.ingenuity.com). To obtain an optimal input gene number for pathway analysis, thresholds of ≥2 (*Scx-Cre; Tsc1* ^*f/f*^) or ≥1.5 (*Scx-Cre; Raptor* ^*f/f*^) fold-change and *p* ≤ 0.05 were used as inclusion criteria for the IPA core analysis. The affected canonical pathways were identified through the IPA statistical algorism based on the built-in knowledge database. The *p* value was calculated by Fischer’s exact test and was adjusted for multiple testing by the Benjamini-Hochberg procedure. Gene Set Enrichment Analysis (GSEA, http://www.gsea-msigdb.org/gsea/index.jsp) was used to identify key genes in RNA-seq datasets. In this study, we used the genes sets including TGF signaling (BIOCARTA_TGFB_PATHWAY (M18933)), FGF signaling (PID_FGF_PATHWAY (M276)), BMP signaling (GO_RESPONSE_TO_BMP (M14241)), and extracellular matrix (GO_EXTRACELLULAR_MATRIX_DISASSEMBLY (M14861) and GO_EXTRACELLULAR_MATRIX_COMPONENT (M17398)). The Heatmap presentation of data was generated using GraphPad Prism 7 (La Jolla, CA).

### Collagen EM analysis

Mouse hindlimbs were fixed in freshly prepared 1.5% glutaraldehyde/1.5% paraformaldehyde (Tousimis) with 0.05% tannic acid (Sigma) in serum-free Dulbecco’s Modification of Eagle’s Medium (DMEM) at 4 °C overnight. Following dissection, samples were post-fixed in 1% OsO_4_, rinsed in DMEM and dehydrated in a graded series of ethanol to 100%. Samples were then rinsed in propylene oxide, infiltrated in Spurrs epoxy, and polymerized at 70 °C overnight. TEM images were acquired using a FEI G20 TEM at multiple magnifications to visualize transverse sections of collagen fibrils. Collagen fibril diameter was measured using Bioquant Osteo II (Nashville, TN).

### qRT-PCR

Achilles tendons were excised from the calcaneus, immediately distal to the tendon-muscular-junction. RNA was extracted with Trizol/choroform and purified with the RNeasy mini kit with on-column DNase I digestion (Qiagen). qRT-PCR was performed on the LightCycler^®^ 96 System (Roche) using gene-specific primers and FastStart SYBR Green I (Roche) following cDNA synthesis with iScript (Bio-Rad). The sequences of primers are listed in Supplementary Table 4.

### Statistical Analysis

Results are presented as mean ± standard deviation. At least three mice per group were analyzed. Statistical significance was determined by Student’s *t*-test with *p* ≤ 0.05 considered as significant unless otherwise indicated.

The datasets generated and/or analyzed during the current study are available from the corresponding author upon reasonable request.

## Electronic supplementary material

Supplementary Figures

Supplementary table 1

supplementary table 2

Supplementary table 3

Supplementary table 4
