# Supplementary material for: mTORC1 Signaling is a Critical Regulator of Postnatal Tendon Development
Source: Sci Rep. 2017 Dec 7;7:17175. doi: 10.1038/s41598-017-17384-0 (PMC5719403; doi:10.1038/s41598-017-17384-0)
Supplement: Supplementary file 1 — Supplementary Figures [file 41598_2017_17384_MOESM1_ESM.pdf]

mTORC1 Signaling is a Critical Regulator of Postnatal Tendon Development

Joohyun Lim[1], Elda Munivez[1], Ming-Ming Jiang[1], I-Wen Song[1], Francis Gannon[2], Douglas R. Keene[3], Ronen Schweitzer[3], Brendan H. Lee[1*], Kyu Sang Joeng[1]

[1]Department of Molecular and Human Genetics, Baylor College of Medicine, One Baylor Plaza, Houston, TX 77030, USA.

[2]Department of Pathology, Baylor College of Medicine, One Baylor Plaza, Houston, TX 77030, USA.

[3]Research Center, Shriners Hospital for Children, Portland, OR 97239, USA.

Supplementary Figures

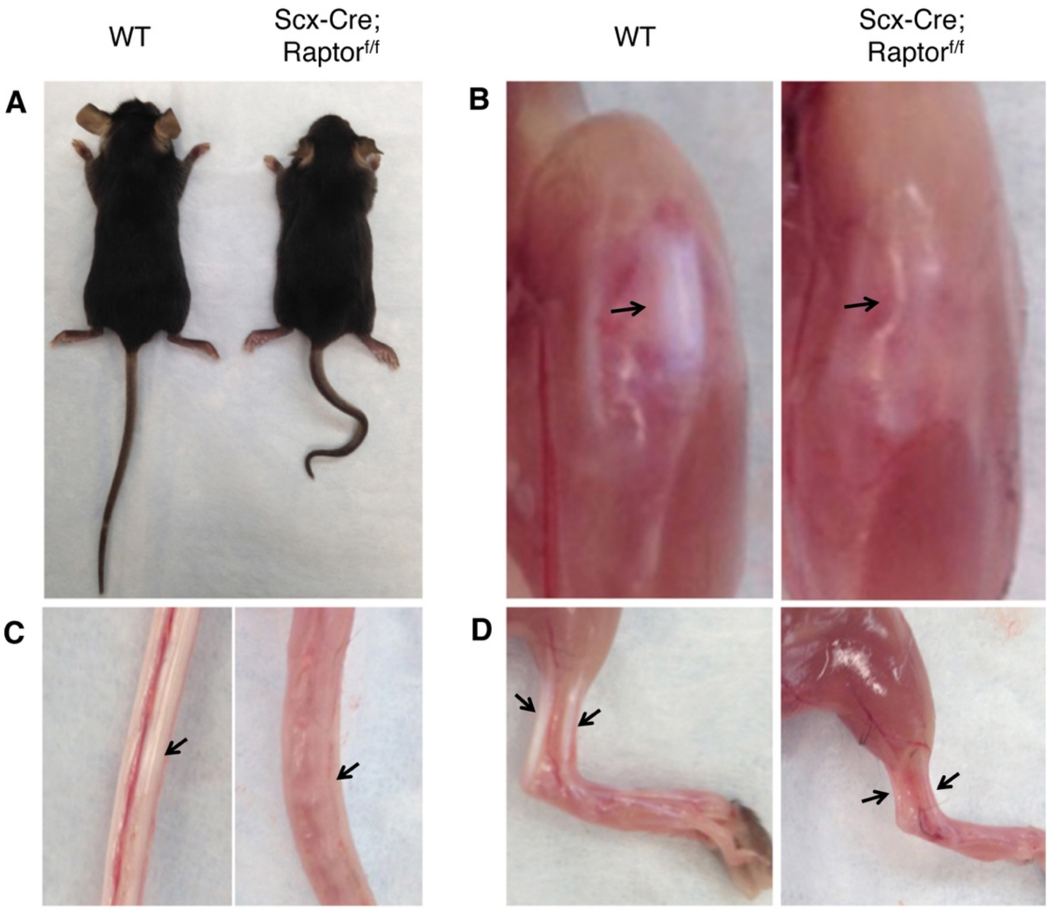

Figure S1. Loss of mTORC1 in tendons caused curly tails and translucent tendons.

(A) The straight tail of wildtype mice and the curly tail of *Scx-Cre; Raptor$^{f/f}$* littermates at 1 month of age. (B-D) Visual examination of patellar (B), tail (C), and Achilles tendon (D) showed white color in wildtype and translucent color in *Scx-Cre; Raptor$^{f/f}$* littermates. Arrows indicate tendons.

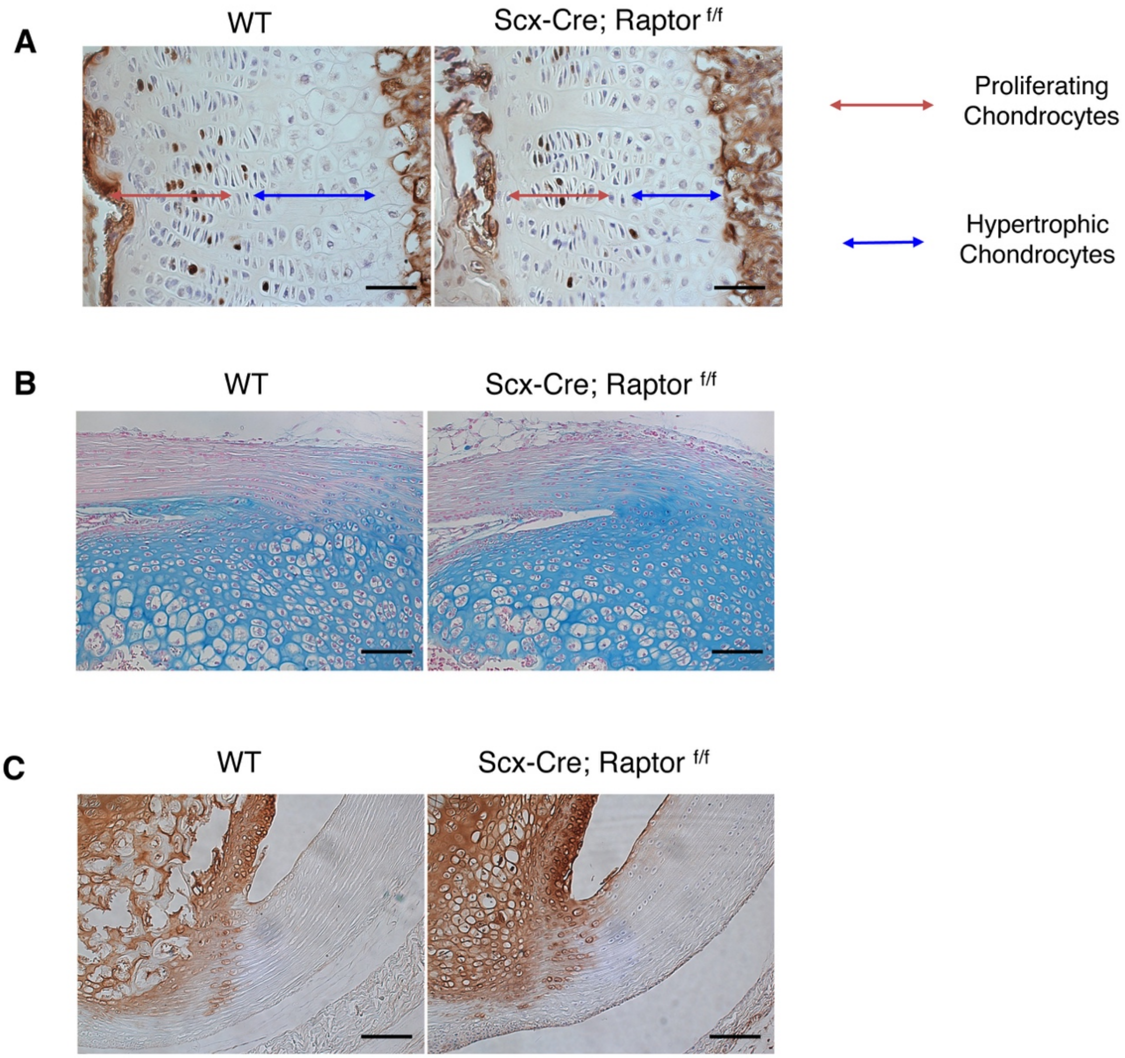

Figure S2. (A) BrdU staining (BrdU) of the tibia growth plate from wildtype and *Scx-Cre; Raptor*$^{f/f}$ littermates at postnatal day 30 (P30). (B) Alcian blue staining of the enthesis of patellar tendon (integration site of patellar to tibia) from wildtype and *Scx-Cre; Raptor*$^{f/f}$ littermates at P30. (C) Col2a1 immunohistochemistry of the enthesis of patellar tendon from wildtype and *Scx-Cre; Raptor*$^{f/f}$ littermates at P30. Scale bars indicate 100μm.

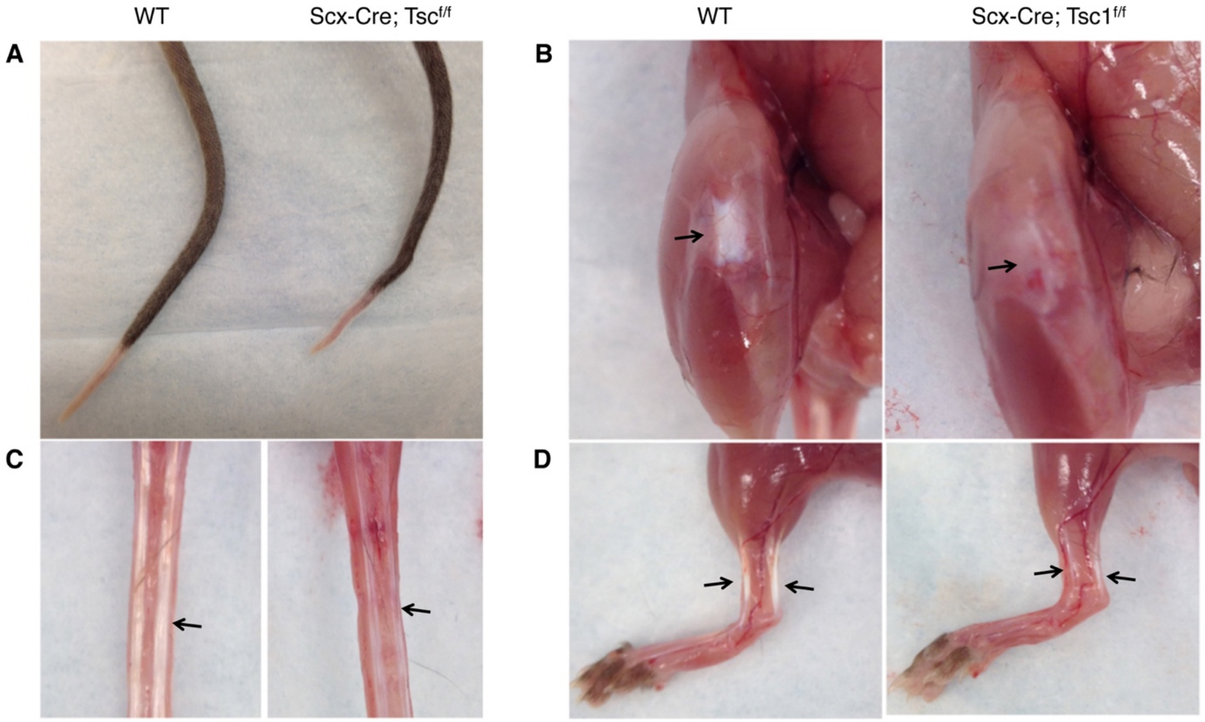

Figure S3. Gain of mTORC1 in tendon caused kinked tails and pale-white tendons.

(A) The straight tail of wildtype mice and kinks at the distal end of the tail of *Scx-Cre; Tsc1*$^{f/f}$ littermates at 1 month of age. (B-D) Visual examination of patellar (B), tail (C), and Achilles tendons (D) in wildtype and *Scx-Cre; Tsc1*$^{f/f}$ littermates. Arrows indicate tendons.

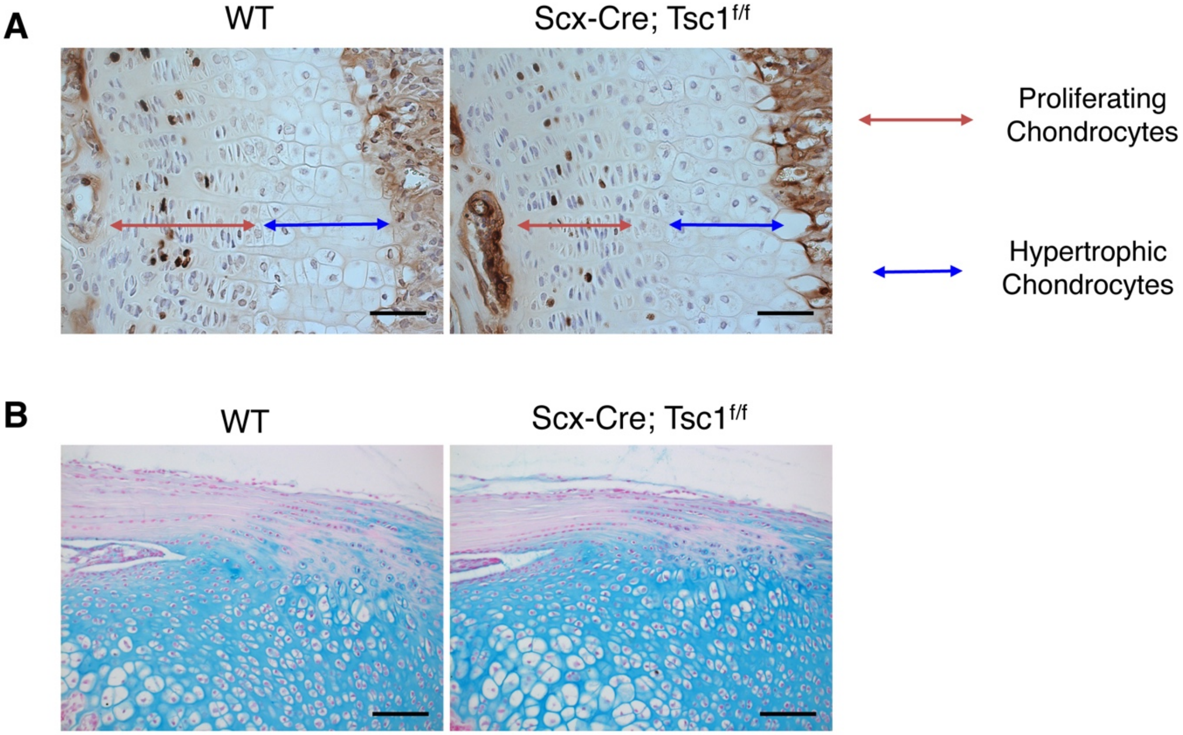

Figure S4. (A) BrdU staining (BrdU) of the tibia growth plate from wildtype and *Scx-Cre; Tsc1^f/f^* littermates at P30. (B) Alcian blue staining of the enthesis of patellar tendon from wildtype and *Scx-Cre; Tsc1^f/f^* littermates at P30. Scale bars indicate 100μm.

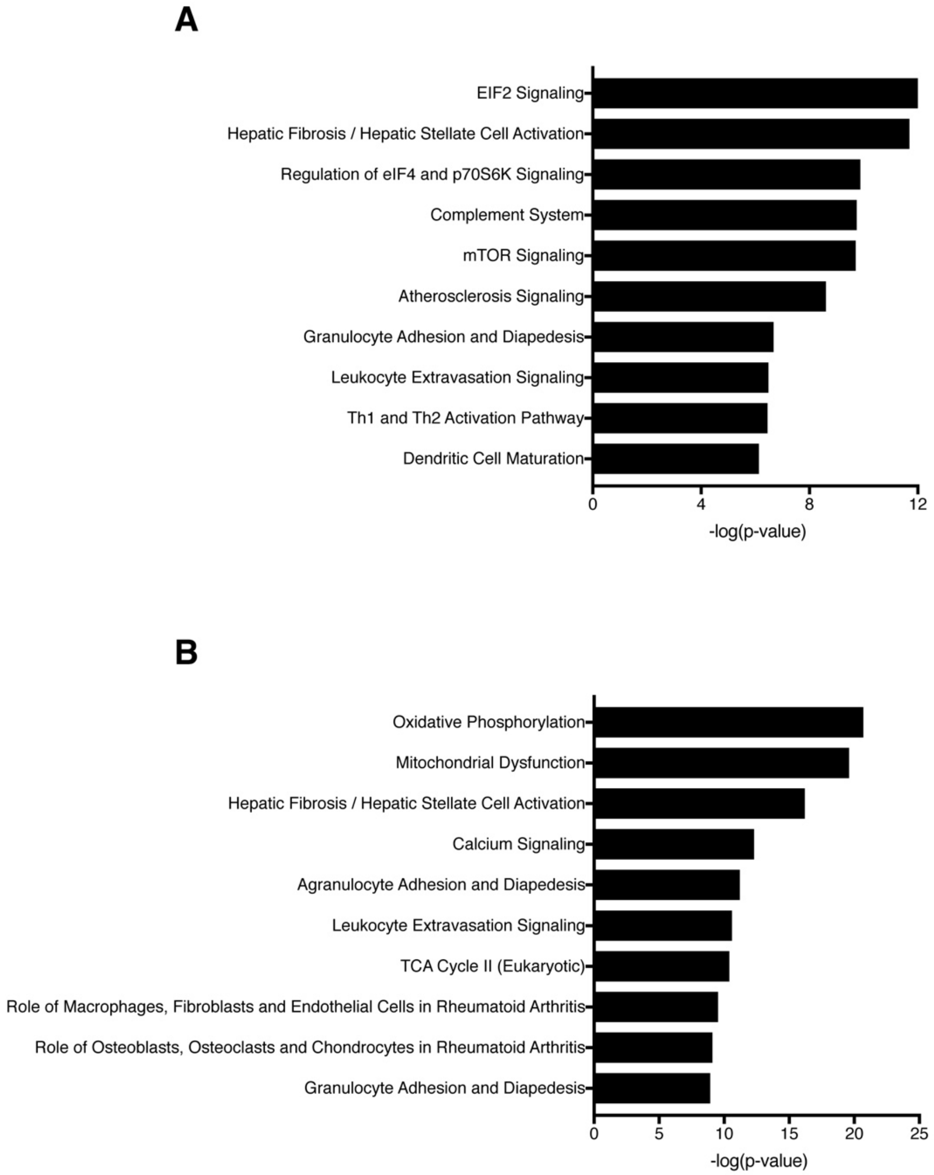

Figure S5. Ingenuity system (IPA) core analysis revealed differential effects of loss- or gain-of mTORC1 signaling. Results of top 10 affected canonical pathways in (A) *Scx-Cre; Raptor*$^{f/f}$ and (B) *Scx-Cre; Tsc1*$^{f/f}$ mice. X-axis: -log(P-value). Y-axis: pathways identified by IPA.
